# Expression and Regulation Profile of Mature MicroRNA in the Pig: Relevance to Xenotransplantation

**DOI:** 10.1155/2018/2983908

**Published:** 2018-03-21

**Authors:** Zongpei Song, David K. C. Cooper, Zhiming Cai, Lisha Mou

**Affiliations:** ^1^Shenzhen Xenotransplantation Medical Engineering Research and Development Center, Institute of Translational Medicine, Shenzhen Second People's Hospital, First Affiliated Hospital of Shenzhen University, Shenzhen University School of Medicine, Shenzhen, Guangdong 518035, China; ^2^Department of Biochemistry, Zhongshan School of Medicine, Sun Yat-sen University, Guangzhou, Guangdong 510080, China; ^3^Xenotransplantation Program, Department of Surgery, University of Alabama at Birmingham, Birmingham, AL 35233, USA

## Abstract

The pig is an important source of meat production and provides a valuable model for certain human diseases. MicroRNA (miRNA), which is noncoding RNA and regulates gene expression at the posttranscriptional level, plays a critical role in various biological processes. Studies on identification and function of mature miRNAs in multiple pig tissues are increasing, yet the literature is limited. Therefore, we reviewed current research to determine the miRNAs expressed in specific pig tissues that are involved in carcass values (including muscle and adipocytes), reproduction (including pituitary, testis, and ovary), and development of some solid organs (e.g., brain, lung, kidney, and liver). We also discuss the possible regulating mechanisms of miRNA. Finally, as pig organs are suitable candidates for xenotransplantation, biomarkers of their miRNA in xenotransplantation were evaluated.

## 1. Introduction

The pig is one of the most widespread livestock species in the world, providing meat production [1]. Although relatively expensive to breed and with a relatively long gestation period (about 114 days) [2], the pig is still an ideal animal model for biomedical research because of its close phylogenetic proximity and similarities with humans, such as organ size, anatomical features, physiology, and organ development, compared with the mouse [3]. The pig has been used as a model to study various issues, such as reproduction [4] and the neuronal system [5], and is employed as a source of organs and cells in xenotransplantation [6].

Despite its apparent importance, knowledge about the pig is still much less than has been accumulated for the mouse and rat, such as genome organization and gene expression regulation. The latest porcine genome reference (Sscrofa 11.1) was shared in the NCBI website (https://www.ncbi.nlm.nih.gov/genome/?term=pig) by the Swine Genome Sequencing Consortium (SGSC) in 2017.

When compared with the human, the pig's genome is of comparable size and contains a similar number of protein-coding genes [7]. One might think that the pig should also have a similar number of microRNAs (miRNAs). However, the number of porcine miRNAs available in public databases is still limited, with only 411 mature miRNAs in the miRBase (v21) compared to human (2,588) and mouse (1,982) [8], partly because only part of the porcine genome is available for study [9]. The common research strategy on miRNA may include three aims: (i) characterization of miRNAs (including identification of novel miRNAs), (ii) determination of target genes, and (iii) illumination of function of miRNAs and target genes. These strategies have been widely used to describe the miRNAome in various pig tissues, such as muscle [9–12], brain [13], fat [14], embryo [3], pituitary [15], intestine [16], ovary [17], and testes [4].

This review is focused on the possible functions and regulating mechanisms of miRNAs in pigs, aiming towards a better understanding of the miRNAome in various tissues. Because of many similarities in morphology and physiology between pig and human, we also evaluate biomarker values of pig miRNA in pig-to-human xenotransplantation.

## 2. Origins and Mechanisms of miRNAs

**Figure 1:**
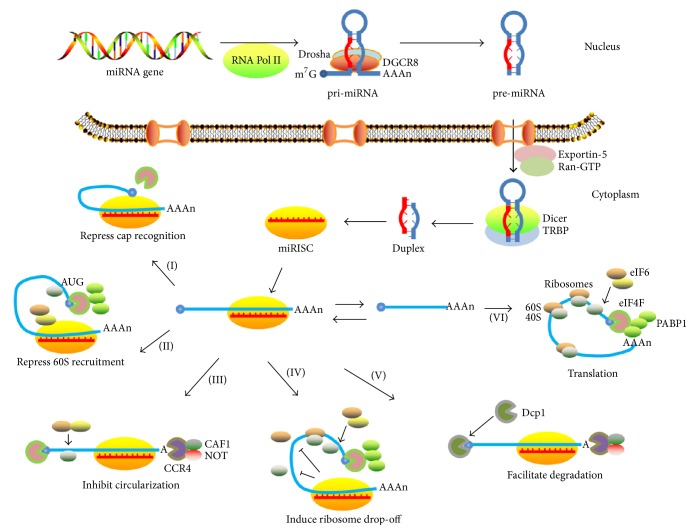
Biogenesis of miRNA and assembly into miRISC and possible mechanisms of miRISC-mediated repression. In animals, the pri-miRNA is transcribed by RNA polymerase II from genomic DNA and is processed by Drosha with the aid of DGCR8 to generate a pre-miRNA species, which is exported from the nucleus and processed by Dicer to form the mature miRNA/miRNA^*∗*^ duplex. Generally, only one strand of the duplex is then assembled into miRISC. When RISCs bind to mRNAs, they can repress initiation of translation at the stage of cap recognition (I) or 60S recruitment (II). Alternatively, they can induce mRNA deadenylation and thereby inhibit its circularization (III). They can also repress translation at the postinitiation stage through inducing ribosomes to dissociate prematurely (IV). They can also induce deadenylation followed by decapping to facilitate mRNA degradation (V). Without repression, mRNAs recruit initiation factors and ribosomal subunits and form circularized structures (VI).

The miRNAs, which are typical transcripts of RNA polymerase II, are small noncoding RNAs in animals and plants [67]. They are transcribed from genomic DNA as long hairpins (pri-miRNA) with an imperfectly paired stem of ~33 bp [68]. The pri-miRNA is excised by Drosha to generate a pre-miRNA species in the nucleus, which is the first processing step. In the second processing step, pre-miRNA is exported from the nucleus and processed by Dicer to form the mature miRNA/miRNA^*∗*^ duplex of ~22 bp length. The miRNA is then assembled into RISC (RNA-induced silencing complexes). Generally, only one strand of the duplex is stably associated with an RISC [69] (Figure 1).

The miRNA acts as an adaptor for RISC to specifically recognize and regulate particular target mRNAs (Figure 1). Recognition involves Watson-Crick base pairing of the 2–8th miRNA nucleotides, which is the so-called seed region [70]. The binding sites of miRNA to mRNAs are located in the 3′ UTR (untranslated region) and usually exist in multiple copies. Most animal miRNAs bind imperfectly with mismatches. When RISCs bind to mRNAs, they can repress initiation of translation at the stage of cap recognition or 60S recruitment [71, 72]. Alternatively, they can induce mRNA deadenylation and thereby inhibit its circularization [73]. They can also repress translation at postinitiation stage through inducing ribosomes to drop off prematurely [74]. They can also induce deadenylation followed by decapping to facilitate mRNA degradation [75, 76] (Figure 1).

## 3. miRNAs in Pig Skeletal Muscle (Table 1)

As the pig is an agriculturally important species, miRNAs that affect development and growth of economically important skeletal muscle are of interest. Several miRNAs may promote myogenesis [18, 19, 23–28] (Table 1). Several others have potential function during muscle development [20–22, 28–41]. Others are involved in the development of the* longissimus dorsi *and* psoas major* muscles [20–22, 39–41], whereas others [9, 42–45] are expressed in a stage-specific manner across muscle development periods. Among them, the miR-1, miR-133, and miR-206, which are specifically expressed in cardiac and skeletal muscle [25], are frequently listed as the highest expressed miRNAs in porcine muscle [9, 10, 34, 77, 78]. These multiple above-mentioned miRNAs provide insights into the regulation of muscle growth and are potential candidates for further improvement of meat quality and production using molecular approaches.

## 4. miRNAs in Pig Adipose Tissue (Table 2)

In addition to skeletal muscle, adipose also affects the carcass value of a pig, including meat quality grade and yield. It also plays an important role in metabolic health. miR-143 was the first miRNA reported to be involved in adipose cell biology [46–48] (Table 2). The miR-210 [14, 50] and the miR-27 family [14, 51] are involved in adipogenesis. Several are abundant in both visceral and subcutaneous adipose tissues [48–50, 52], whereas others are subcutaneous adipose tissue-specific enriched miRNAs [52–55]. Some are specifically expressed in the greater omentum [52, 56, 57]. Research into pig adipose tissue miRNAs may be beneficial in meeting the increasing demand of consumers for improved pork quality, which is a topic of worldwide concern [14].

## 5. miRNAs in Other Pig Tissues (Table 3)

Although most research has hitherto been dedicated to miRNA's roles in meat quality, emerging research has evaluated miRNA in other solid tissues in pigs, including, but not limited to, reproduction. Many miRNAs are enriched in reproductive tissues [4, 15, 17, 37] (Table 3). Others may play a role in differentiating neurons in brain development [3, 13, 58–61]. Several are highly expressed in big solid organs [1, 37, 62–64]. The miR-200b and miR-214 are key miRNAs in tooth development [65, 66].

When a miRNA is predominant, this suggests that it could have a significant role in the tissue and that it could govern or be implicated in the major constitutive functions carried out by this tissue.

## 6. Biomarker Values of miRNA in Xenotransplantation

The pig has become the most suitable candidate as a source organ for xenotransplantation to overcome the growing gap between the need and availability of human donor organs [6]. Detailed genome information and emerging gene-editing technologies increase the possibility of producing pigs specific for this purpose. The xenotransplantation of organs from gene-modified pigs is associated with longer survival and less rejection [6]. Biopsy is the gold standard for diagnosis of conditions such as acute rejection (AR), disease recurrence, and drug toxicity [79]. However, biopsy often relies on “subjective” measures, with some variability in results and reporting methods among pathologists, or limited diagnostic accuracy associated with sampling error [80]. There is a critical need for biomarkers for early diagnosis, treatment response, and outcome prediction in organ transplantation, with the final goal of an individualized treatment to prevent or reverse graft injury [81].

The miRNAs may be ideal candidates as biomarkers of disease [82–86]. Several factors (the relatively consistent changes seen in diseases, reliable analysis methods, tissue-specific expression patterns, less complexity than mRNAs, no postprocessing modification, and amplifiable signals) contribute to making miRNAs ideal candidates, especially in the cancer diagnostic field [87–91]. Profiling miRNAs can be used as markers of organ donor quality/ischemia reperfusion injury [92]. The strong association between miRNA expression and allograft function or acute rejection demonstrates that miRNAs may be excellent biomarkers of human allograft status [81, 93]. For example, levels of miR-142-5p, miR-155, and miR-223 can each predict acute rejection with >90% sensitivity and specificity in human renal allografts [94, 95]. Because miRNAs are stably expressed in serum, plasma, urine, saliva, and other body fluids, this makes them ideal noninvasive biomarkers [83] to accurately monitor graft function in xenotransplantation. There are minor differences in the nucleotide composition of miRNAs among species [62]. Therefore, the circulating pig-specific miRNAs in human body fluids have vast potential to be biomarkers after pig-to-human xenotransplantation. Graft tissue and/or circulating miRNA profiles may be used as new biomarkers in guiding the diagnostic, therapeutic, and prognostic strategies that are associated with overimmunosuppression, organ toxicity, and graft rejection or loss.

## 7. Discussion

Knowledge of human development, physiology, and pathology can be obtained from suitable animal models, especially the mouse and rat, but many of their physiological parameters (e.g., size, respiratory rate) are significantly different from those in humans [2]. Rodent genomes also have a faster rate of evolution than the human genome [96]. The pig is not only of significant agricultural value but is also considered a good model for biomedical research [97]. Furthermore, pigs have been identified as the most promising source of organs for xenotransplantation to counteract the shortage of human organs for transplantation [98].

Many miRNAs are highly conserved among related species [99]. Studies on miRNAs in the pig will be beneficial in understanding their key regulatory roles in humans. To obtain a better insight into the biological functions of miRNAs, it is imperative to identify all miRNAs expressed in the pig genome and their potential mRNA targets [100, 101], which is becoming easier using bioinformatic methods, with a growing number of excellent tools becoming available [8, 102]. However, the false discovery rate in predictive results remains high, and experimental validation will be needed after bioinformatic prediction. We suggest, however, that pig miRNA profiles will be used as new biomarkers in pig-to-human xenotransplantation.

## Figures and Tables

**Table 1:** miRNAs in pig skeletal muscle.

miRNA	Target	Function	Reference
miR-1a	HDAC4	It promotes myogenesis during embryonic development and muscle cell differentiation	[18–22]

miR-133	SRF	It enhances the proliferation of myoblasts	[19–22]

miR-27b		It is involved in myogenic differentiation, fast-specific and glucocorticoid-dependent myostatin expression	[23–25]

miR-148a	ROCK1	A novel myogenic miRNA that mediates myogenic differentiation	[26]

miR-143		It controls performance of different fiber types	[27, 28]

miR-378	BMP2	A candidate for myogenesis	[29, 30]
	MAPK1		

miR-128		It regulates adipogenesis, osteogenesis, and myogenesis	[31]

miR-126		It attenuates insulin signaling and governs vascular integrity and angiogenesis	[32, 33]

miR-92a		It regulates skeletal muscle growth	[28]

miR-127		It regulates the callipyge muscular hypertrophy phenotype	[34, 35]
miR-432			
miR-136			

miR-10b		It regulates myogenesis and muscle development	[34, 36]

let-7 family		Key miRNA regulators of development	[37, 38]

miR-103		It is involved in cellular Acetyl-CoA and lipid levels	[20, 39]
miR-107			

miR-23	PGC-1*α*	It affects the ratio of oxidative red muscle and oxidative white muscle fibers	[20, 40, 41]

miR-181	Hox-A11	It is barely detectable in resting muscle and establishes the muscle phenotype	[42, 43]

miR-206	Cx43	It is only highly expressed in newly formed muscle fibers and promotes myoblast differentiation and development	[25, 44, 45]

miR-486		It is expressed postnatally, acts as an inhibitor of myogenesis	[9]

miR-376b		expressed prenatally, and plays a role in promotion of myogenesis	[9]

miR-363 miR-365 miR-422b		They are differentially expressed between 33 days postgestation and adult life, long-term regulation of muscle growth and development	[9]

**Table 2:** miRNAs in pig adipose tissue.

miRNA	Target	Function	Reference
miR-143	ERK5	It promotes adipocyte differentiation	[46–49]

miR-210		It promotes adipogenesis	[14, 50]

miR-27 family	INSR IRS1–4 PDK1/2 CREB S6K1	It inhibits adipogenesis	[14, 51]

miR-148a-3p		It is involved in differentiation of 3T3-L1 preadipocytes	[50]

let-7a-1-5p let-7f-5p		They play potential housekeeping roles in adipocytes	[52]

miR-155-5p	C/EBP-b	It inhibits adipogenesis	[52–55]

miR-193b-3p miR-365		They act as central regulators of brown fat differentiation and adipogenesis	[52]

miR-374b-5p	C/EBP-b	It is involved in the effect of maternal dietary protein on lipid metabolism	[52]

miR-18a-3p miR-20-3p miR-19b-1-5p miR-181a-2-3p miR-181b-2-3p		They are involved in development and production of proinflammatory B-cells and T-cells	[52, 56, 57]

**Table 3:** miRNAs in other pig tissues.

miRNA	Tissue	Function	Reference
miR-7	Pituitary		[15]

miR-760 miR-1296 miR-137 miR-362	Pituitary		[37]

miR-153 miR-205	Mature testis		[4]

miR-196 miR-149^*∗*^ miR-485-3p	Immature testis		[4]

miR-21-5p	Ovary, testis		[17]

miR-9 miR-30a	Head region	miR-9 regulates proliferation and migration of human neural progenitor cells	[3, 58]

miR-17 miR-106a	Neurons and brain	It is involved in neurons differentiation and brain development by regulating APP	[13, 59]

miR-29c	Adult cortex, cerebellum	It is an effective biomarker of radiation-induced brain response	[60, 61]

miR-320	Lung		[62]

miR-375	Stomach and lymph nodes		[62]

miR-23a miR-125b miR-23b miR-126 miR-200b-3p	Kidney		[1]

miR-122-5p	Liver	It plays a role in cholesterol, fatty acid, and lipid metabolism	[37, 63, 64]

miR-200b miR-214	Teeth	miR-200b is key in tooth development	[65, 66]

## Abbreviations

miRNA: MicroRNA
RISC: RNA-induced silencing complexes.
